# Supplementary material for: Brain Transcriptional Profiles of Male Alternative Reproductive Tactics and Females in Bluegill Sunfish
Source: PLoS One. 2016 Dec 1;11(12):e0167509. doi: 10.1371/journal.pone.0167509 (PMC5132329; doi:10.1371/journal.pone.0167509)
Supplement: S1 Fig — (PDF) [file pone.0167509.s001.pdf]

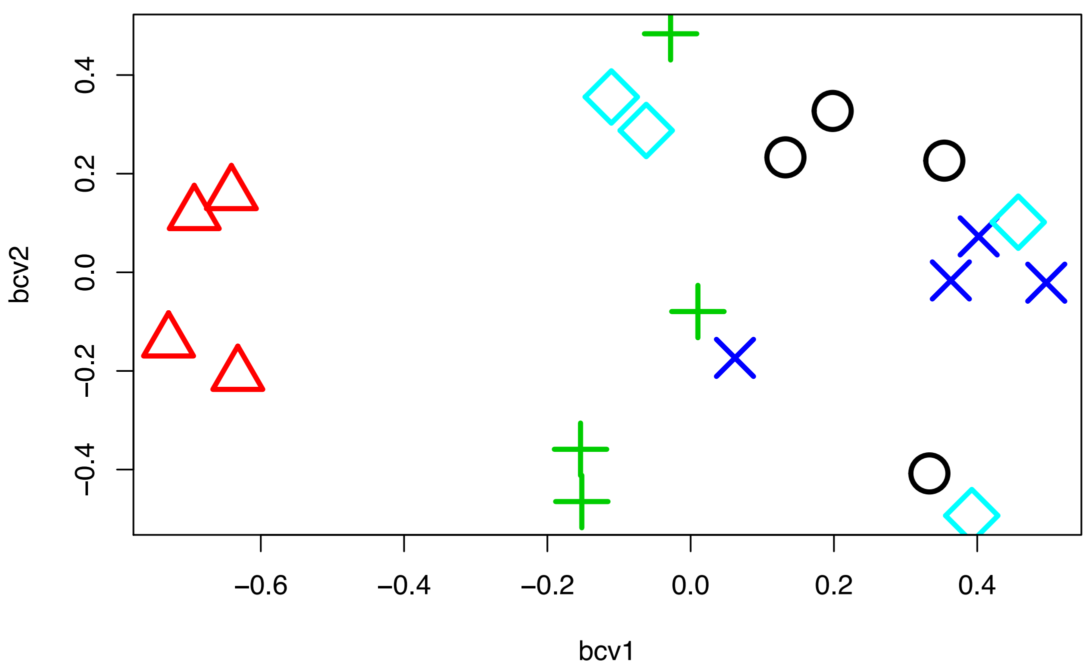

Supplement Figure 1: Multi-dimensional space (MDS) plot based on the biological coefficient of variation (bcv) among bluegill male ARTs. Red triangles: sneaker males, green pluses: satellite males, black circles: spawning parental males, blue X: non-spawning parental males, light blue diamonds: females.
